# MERGING AN AGE FRIENDLY UNIVERSITY WITH AN AGE FRIENDLY HEALTH CARE SYSTEM: CASE STUDY FINDINGS

**DOI:** 10.1093/geroni/igac059.2142

**Published:** 2022-12-20

**Authors:** John Schumacher, Nicole Brandt, Barbara Resnick, Anissa Nahabedian, Mojdeh Heavner, Giora Netzer, Lara Wilson, Raya E Kheirbek

**Affiliations:** University of Maryland, Baltimore County, Baltimore, Maryland, United States; University of Maryland, Baltimore, Baltimore, Maryland, United States; University of Maryland, Baltimore, Maryland, United States; University of Maryland, Baltimore, Baltimore, Maryland, United States; University of Maryland School of Pharmacy, Baltimore, Maryland, United States; University of Maryland, Baltimore, Maryland, United States; University of Maryland Shore Regional Health, Chestertown, Maryland, United States; University of Maryland School of Medicine, Baltimore, Maryland, United States

## Abstract

Age Friendly Health Systems (AFHSs) and Age Friendly Universities (AFUs) are distinct entities in the “Age Friendly” ecosystem. While Age Friendly entities function independently, they typically exist in close proximity (e.g., universities and community hospitals); yet they remain isolated in their Age Friendly efforts. We report on a collaboration between a mid-Atlantic Age Friendly University and a new AFHS using case study methodology. Our goal is to inform and inspire key stakeholders responsible for creating innovative healthy aging communities. The collaboration began with a shared stakeholder team who articulated focus areas and overlapping goals. A charter document was developed articulating commitments and responsibilities. Using a Quality Improvement (QI) approach, projects targeted the hospital’s older patient needs that linked to the AFHS 4 M’s of Matters, Medications, Mobility, and Mentation. University graduate students and faculty volunteered to teach and mentor hospital staff on the QI projects: 1) Get to Know Me Boards filled by staff caring for hospitalized older adults (Matters); 2) Medical Intensive Care Unit discharge opioid medication deprescribing (Medication); 3) UMove Mobility Screening addressing functional status (Mobility); 4) UB-2 Delirium Screening (Mentation). Data collection across projects demonstrated proof-of-concept and identified implementation challenges around communication, screening, data entry, and data extraction from electronic medical records. During Covid-19 pandemic, the collaboration allowed QI projects to conduct multiple Plan-Do-Check-Act cycles while contributing to the Age Friendly goals of both organizations. Partnerships between academic institutions and hospitals foster development of evidence- based healthy aging communities and provide opportunities for continuing education and research.

